# Repeatability of AI-based, automatic measurement of vertebral and cardiovascular imaging biomarkers in low-dose chest CT: the ImaLife cohort

**DOI:** 10.1007/s00330-024-11328-9

**Published:** 2025-01-08

**Authors:** Iris Hamelink, Marcel van Tuinen, Thomas C. Kwee, Peter M. A. van Ooijen, Rozemarijn Vliegenthart

**Affiliations:** 1 Department of Radiology, University of Groningen, University Medical Center of Groningen, Groningen, The Netherlands; 2 Department of Radiation Oncology, University of Groningen, University Medical Center of Groningen, Groningen, The Netherlands; 3 Data Science in Health (DASH), University of Groningen, University Medical Center of Groningen, Groningen, The Netherlands

**Keywords:** Aortic aneurysm (thoracic), Artificial intelligence, Coronary artery disease, Chest CT, Thoracic vertebrae

## Abstract

**Objective:**

To evaluate the repeatability of AI-based automatic measurement of vertebral and cardiovascular markers on low-dose chest CT.

**Methods:**

We included participants of the population-based Imaging in Lifelines (ImaLife) study with low-dose chest CT at baseline and 3–4 month follow-up. An AI system (AI-Rad Companion chest CT prototype) performed automatic segmentation and quantification of vertebral height and density, aortic diameters, heart volume (cardiac chambers plus pericardial fat), and coronary artery calcium volume (CACV). A trained researcher visually checked segmentation accuracy. We evaluated the repeatability of adequate AI-based measurements at baseline and repeat scan using Intraclass Correlation Coefficient (ICC), relative differences, and change in CACV risk categorization, assuming no physiological change.

**Results:**

Overall, 632 participants (63 ± 11 years; 56.6% men) underwent short-term repeat CT (mean interval, 3.9 ± 1.8 months). Visual assessment showed adequate segmentation in both baseline and repeat scan for 98.7% of vertebral measurements, 80.1–99.4% of aortic measurements (except for the sinotubular junction (65.2%)), and 86.0% of CACV. For heart volume, 53.5% of segmentations were adequate at baseline and repeat scans. ICC for adequately segmented cases showed excellent agreement for all biomarkers (ICC > 0.9). Relative difference between baseline and repeat measurements was < 4% for vertebral and aortic measurements, 7.5% for heart volume, and 28.5% for CACV. There was high concordance in CACV risk categorization (81.2%).

**Conclusion:**

In low-dose chest CT, segmentation accuracy of AI-based software was high for vertebral, aortic, and CACV evaluation and relatively low for heart volume. There was excellent repeatability of vertebral and aortic measurements and high concordance in overall CACV risk categorization.

**Key Points:**

***Question*** *Can AI algorithms for opportunistic screening in chest CT obtain an accurate and repeatable result when applied to multiple CT scans of the same participant?*

***Findings*** *Vertebral and aortic analysis showed accurate segmentation and excellent repeatability; coronary calcium segmentation was generally accurate but showed modest repeatability due to a non-electrocardiogram-triggered protocol.*

***Clinical relevance*** *Opportunistic screening for diseases outside the primary purpose of the CT scan is time-consuming. AI allows automated vertebral, aortic, and coronary artery calcium (CAC) assessment, with highly repeatable outcomes of vertebral and aortic biomarkers and high concordance in overall CAC categorization.*

## Introduction

Computed tomography (CT) data from lung cancer screening offers the possibility for simultaneous detection of other subclinical thoracic diseases, among which are musculoskeletal disorders and cardiovascular disease [1]. The introduction of low-dose chest CT screening will result in an increasing number of CT examinations, which poses major challenges for radiologists. Multiple studies have shown that an increased workload and less time allocated for each case contribute to higher error rates and potentially underreporting of findings on CT [2, 3].

Artificial intelligence (AI) has the potential to reduce the workload for radiologists through the introduction of automatic processing of chest CT for lung cancer screening. The automatic detection of lung nodules has previously been investigated and is entering the clinical realm [4, 5]. In addition, AI algorithms have been developed to automatically detect and accurately measure other biomarkers for subclinical diseases on chest CT scans acquired for the purpose of lung cancer screening [6]. This could potentially enable opportunistic screening in a broader spectrum of the normal population, for example of osteopenia based on vertebral height and density, thoracic aortic dilatation and aneurysms based on aortic diameters, and subclinical coronary artery disease based on coronary artery calcium scoring. Yacoub et al (2022) evaluated commercial AI software and showed superior diagnostic performance of AI compared to a radiologist for binary reporting of aortic dilatation and coronary artery calcification in 100 patients with non-contrast chest CT [7]. Previous research [6, 7] mostly focused on the comparison of human reading with AI outcomes. It is yet unclear if AI algorithms are also able to obtain an accurate and repeatable result when applied to multiple CT scans of the same participant. Therefore, we aimed to quantify the segmentation accuracy and repeatability of AI software for automatic measurement of imaging biomarkers on chest CT scans, including vertebral height and density, thoracic aortic diameter at guideline-compliant measurement positions, heart volume, and coronary artery calcification volume (CACV).

## Materials and methods

### Study population and CT acquisition

We performed a retrospective analysis of a prospective study in a subset of participants from the Imaging in Lifelines (ImaLife) study. The aim of ImaLife was to evaluate the distribution of quantitative imaging biomarkers for early stages of lung cancer, chronic obstructive pulmonary disease and cardiovascular disease in a general population. The medical ethics committee of the University Medical Center Groningen (UMCG), the Netherlands, approved the ImaLife study, and all participants provided informed consent. The ImaLife study was registered with the Dutch Central Committee on Research Involving Human Subjects (https://www.toetsingonline.nl, NL58592.042.16). CT scan acquisition included 12,128 participants as representative of the general Northern European population aged 45 years and older.

Participants underwent a non-triggered, non-contrast low-dose chest CT at high pitch on a third-generation dual-source CT system (SOMATOM Force, Siemens Healthineers) between August 2017 and May 2022. The scanning protocol included the following parameters: 120 kVp, 20 mAs, pitch 3.0 (2.5 in large habitus), 1.0/0.7 mm slice thickness. Images were reconstructed with a soft tissue kernel (Br40). Scan acquisition was in supine position and participants were coached to perform breath hold at maximum inspiration [8].

As part of the ImaLife study, 633 participants with intermediate-sized lung nodules (volume, 100–300 mm³) at baseline, underwent a follow-up scan to study natural evolution. The repeat scan was performed 3–4 months after the baseline scan (mean interval, 3.9 ± 1.8 months).

### Automatic measurements by AI

The baseline and repeat chest CT scans for each participant were reviewed by AI software, the AI-Rad Companion chest CT research application (prototype, version 01/2023, Siemens Healthineers). The software automatically generated segmentations for the thoracic vertebrae, aorta, heart (pericard) and coronary artery calcifications (CAC). In addition, it produced guideline-compliant measurements for vertebral height and vertebral density, thoracic aorta diameter, heart volume and CACV as measure of CAC.

A trained, licensed technical physician (I.H.) visually reviewed all AI-Rad Companion assessments for accuracy of segmentations of the thoracic vertebrae (T1-T12), aorta (including individual positioning according to guidelines [9, 10]), pericardium and coronary calcium. The reader was supervised by a board-certified ECBR radiologist with 15+ years of experience (R.V.) for inconclusive cases. All segmentations were checked according to the following qualitative grading: accurate, suboptimal, inaccurate. Suboptimal cases included small errors made by AI, but which were not deemed clinically significant. Further details on our grading system are provided in Supplementary Table 1. Visual examples of accurate, suboptimal and inaccurate segmentation are provided in Supplementary Fig. 1. Vertebral analysis was checked for accurate segmentation and position of height measurement at vertebral level, accurate numbering of the vertebrae and accurate placement of the regions of interest (ROIs) from which vertebral density was determined. Only segmentation errors were considered as impactful for quantitative outcomes, not thoracic vertebra numbering errors. Aortic measurements were evaluated regarding accuracy of segmentation, positioning and angulation of the guideline-compliant diameter measurements [9, 10] at the position of the sinotubular junction, mid ascending aorta, proximal aortic arch, mid aortic arch, proximal descending aorta, mid descending aorta, and aorta at diaphragm level. Measurements at the sinus of Valsalva and the abdominal region were excluded from analysis, due to the relatively high discrepancy compared to human readers in a previous study [11] and the absence of imaging in the abdominal region in a large part of the chest CT scans, respectively. Accuracy of heart volume was determined by accurate segmentation of the pericardium. CACV was checked for accurate segmentation of coronary calcifications. CACV, influenced by motion but correctly segmented by AI, was deemed accurate. The cases were reviewed in random order and the reviewer was blinded to AI measurements of the same participant in the baseline scan while reviewing the AI measurements for the repeat scan (and vice versa). No adjustments or corrections were made to the AI segmentations and measurements. It was assumed that between scans, no physiological change occurred outside the nodule dynamics. It was visually verified no vertebral fractures occurred during the interval between baseline and repeated CT. For quantification of CACV, participants with a history of percutaneous coronary intervention (PCI) or coronary artery bypass grafting (CABG) were excluded from the analysis.

### Statistical analysis

For the primary analysis, only cases that were visually assessed as adequately segmented (accurate: yes or suboptimal) in both baseline and repeat scan were included for further quantitative analysis including Intraclass Correlation Coefficient (ICC) and assessment of relative difference in measurements.

Continuous variables for vertebral, aortic and heart analysis were represented as mean ± standard deviation (SD). Continuous variables for CACV were represented as median values accompanied by the interquartile range, due to the non-normal distribution of CACV. Agreement between baseline and repeat measurements was expressed using the ICC and corresponding 95% confidence interval (95% CI). An ICC > 0.9 was considered excellent, 0.75–0.9 was considered good, 0.5–0.75 moderate, and < 0.5 indicated poor agreement [12]. Relative difference was defined as 100 times the absolute difference between baseline and repeat value divided by the mean value of baseline and repeat values. For scans where one of the two measurements was visually assessed as inadequate, we performed a supplemental analysis, where the difference between the ‘inadequate’ value and the ‘adequate’ value was divided by the ‘adequate’ value (instead of the mean value). CACV-based risk stratification in strata proposed by Mets et al [13] (< 10; 10–99; 100–499; ≥ 500) was compared for baseline and repeat CT and evaluated by percentages of concordance. The reliability of risk classification was assessed with Cohen’s kappa statistics, with a *p*-value of < 0.001 for statistical significance. All statistical analyses were performed using SPSS version 28.0.1.0 (SPSS, Inc.).

## Results

### Participant characteristics and AI analysis

Of 12,128 ImaLife participants, 633 received repeat chest CT scanning. One participant was excluded due to corrupt CT data, resulting in 632 participants (63.3 ± 11.0 years, 56.6% men, mean body mass index 26.3 ± 4.0 kg/m²) being included for analysis. AI-based vertebral analysis was complete in all participants. In 8 participants, AI-based aortic analysis was incomplete in either baseline or repeat scan, resulting in 624 participants for visual analysis. AI-based heart analysis was complete in 630 cases. AI-based CACV scoring was complete for 605 participants (*n* = 26 excluded due to PCI/CABG participants, *n* = 1 due to incomplete AI analysis).

### Visual assessment

**Fig. 1:**
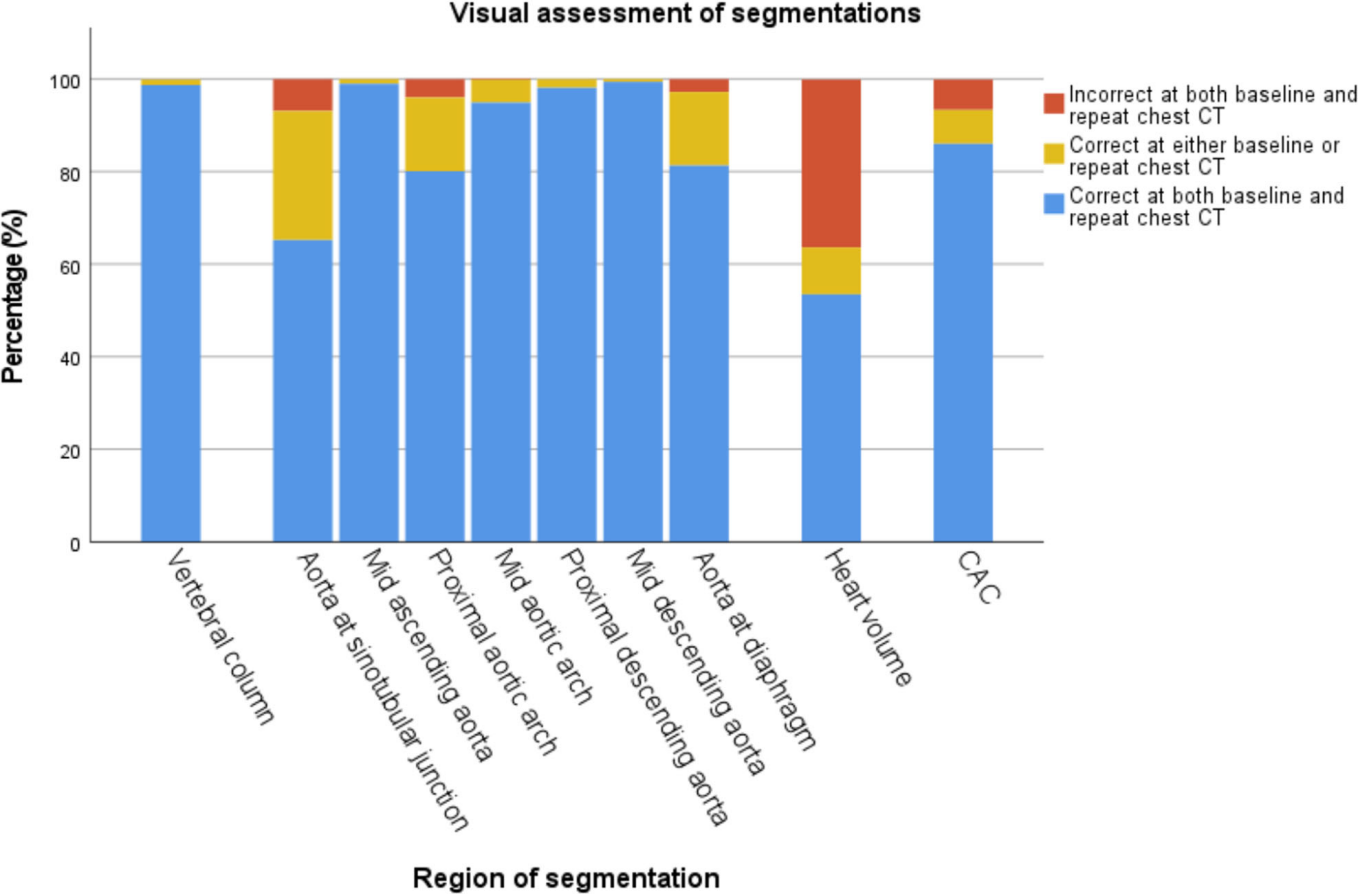
Results of visual assessment indicating percentage of participants in which AI segmentations were assessed as correct in both, either or neither of baseline and repeat chest CT scan. Segmentation accuracy was high (> 80%) for all biomarkers, except for thoracic aortic diameter measurements at the sinotubular junction (66.1%) and heart volume (53.5%). CAC, coronary artery calcium

**Fig. 2:**
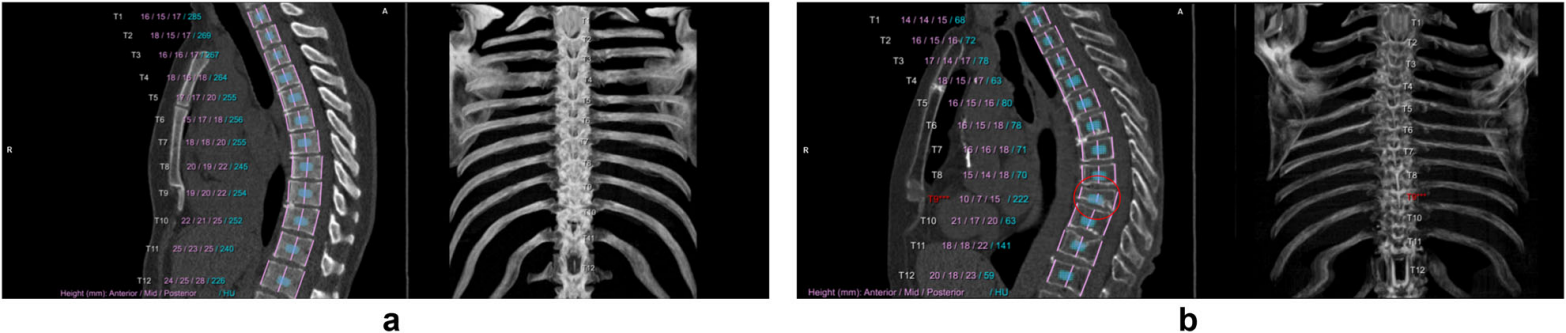
**a** Correct AI-based vertebral analysis and (**b**) inaccurate AI-based vertebral analysis due to incorrect segmentation of T9 (circled, red). The pink lines represent height measurements and the blue ROIs for density measurements

**Fig. 3:**
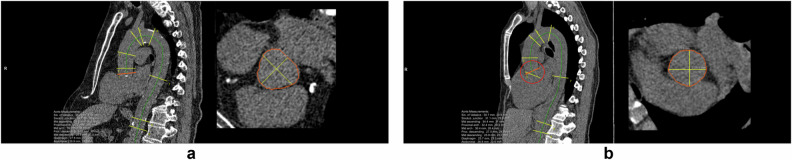
**a** Accurate AI-based measurement of the thoracic aortic diameter along the entire thoracic aorta and **b** incorrect aortic measurement at the level of the sinotubular junction due to incorrect plane alignment (circled, red). The green line represents the centerline, yellow and red lines are the individual measurement locations aligned along the aorta

**Fig. 4:**
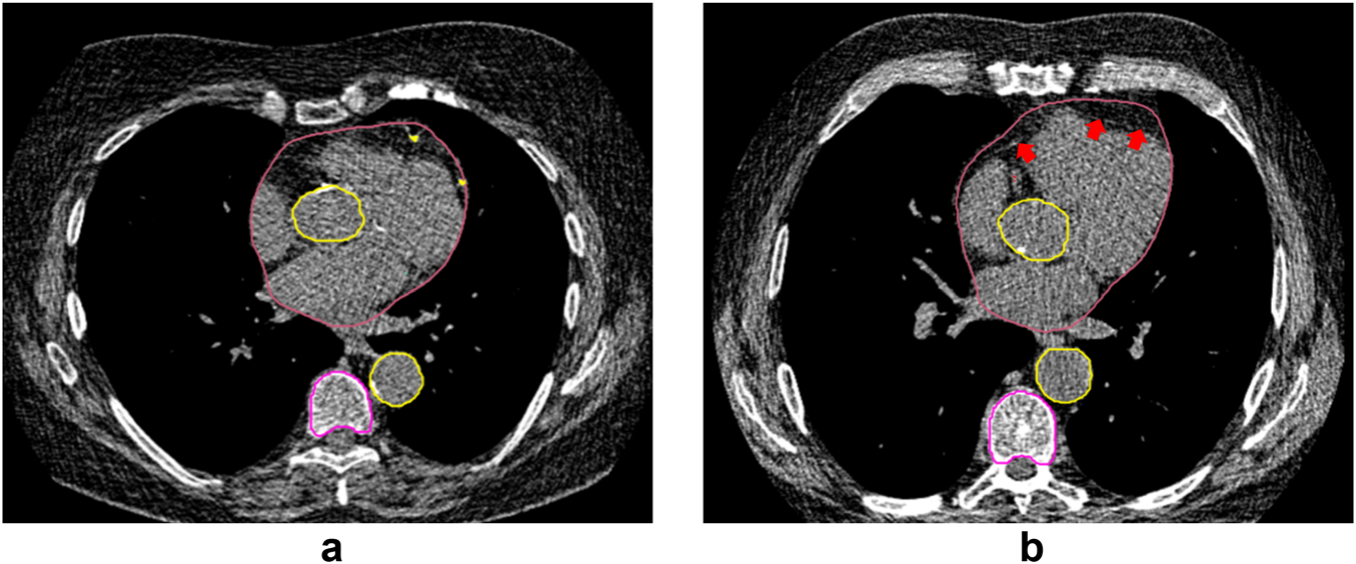
**a** Correct segmentation of the pericardium (red). **b** Overestimation of the heart volume caused by incorrect pericardial segmentation including paracardial fat. The red arrows are aligned with the actual pericardium

**Fig. 5:**
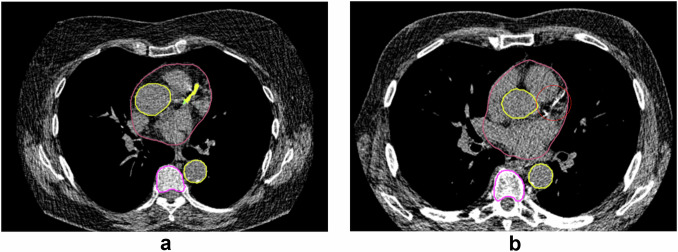
**a** Correct AI-based CACS Green and yellow segmentation represent calcifications at the position of the left main and left anterior descending coronary arteries. **b** Inaccurate AI-based CACS due to incorrect segmentation of calcification in the left main and left anterior descending coronary arteries (circled, red)

Results of the visual assessment of AI-based measurements are shown in Fig. 1. In Supplementary Fig. 1, examples classified as suboptimal are shown to clarify the categorization of visual assessments. Vertebral and aortic analysis showed the highest segmentation accuracy. There was inadequate segmentation of the vertebral column in 8 participants and inaccurate numbering of the vertebrae in 95 participants in baseline or repeat scan. Height and density measurement of T1 was incomplete in 21.5% of included chest CT scans and therefore excluded from further quantitative analysis. AI-based analysis at the mid ascending and the mid descending thoracic aorta resulted in > 99% of measurements classified as adequate at baseline and repeat scan. Diameter measurement at the sinotubular junction showed the lowest accuracy due to non-optimal or incorrect angulation of the measurement plane in 66.1% of inadequate cases. Visual assessment of heart volume segmentation showed a relatively low accuracy, with only 53.5% of segmentations adequate at baseline and follow-up. The main reason for failure was incorrect tracing of the thin pericardium, leading to inclusion of paracardial fat within the volume and resulting in overestimation of the heart volume. AI-based CAC segmentation was considered adequate in 520 participants (86.0%) in both baseline and repeat scan. CAC segmentation mainly failed within the left main and left anterior descending coronary arteries, ignoring large calcifications at that site. Other reasons for inadequate CACV were the inclusion of mitral valve calcification as CAC and inadequate segmentation of right CAC. Figures 2 to 5 show examples of accurate and inaccurate segmentation of imaging biomarkers by the AI software. Only measurements visually assessed as adequate (accurate or suboptimal) in both baseline and repeat scan were included for further analysis including ICC and assessment of relative difference. The final number of repeated scans included per biomarker is reported in Table 1.

**Table 1:** AI-based quantitative assessment of vertebral and cardiovascular imaging biomarkers in low-dose chest CT, mean value at baseline and absolute/relative difference between baseline and repeat CT for those with accurate segmentation on both CT scans

	Baseline value	Absolute difference	Relative difference (%)
Vertebrae (*N* = 624)			
Mean height diameter mean ± SD* (mm)	20.3 ± 1.3	0.2 ± 0.2	1.0 ± 1.1
Anterior height diameter mean ± SD (mm)	19.8 ± 1.3	0.3 ± 0.3	1.6 ± 1.5
Middle height diameter mean ± SD (mm)	19.1 ± 1.3	0.3 ± 0.3	1.6 ± 1.4
Posterior height diameter mean ± SD (mm)	21.9 ± 1.5	0.3 ± 0.3	1.5 ± 1.4
Density mean ± SD (HU)	145.1 ± 38.7	3.8 ± 3.1	2.8 ± 2.5
Thoracic aorta (*N* = 407–620)			
Sinotubular junction (*N* = 407) mean ± SD (mm)	33.4 ± 3.3	0.7 ± 0.6	2.0 ± 1.7
Mid ascending (*N* = 618) mean ± SD (mm)	37.2 ± 4.0	0.7 ± 0.6	1.9 ± 1.6
Proximal arch (*N* = 500) mean ± SD (mm)	33.9 ± 3.3	0.8 ± 0.6	2.3 ± 1.9
Mid arch (*N* = 592) mean ± SD (mm)	31.1 ± 2.7	0.6 ± 0.5	2.1 ± 1.7
Proximal descending (*N* = 612) mean ± SD (mm)	29.5 ± 3.0	0.7 ± 0.6	2.5 ± 2.0
Mid descending (*N* = 620) mean ± SD (mm)	27.4 ± 2.9	0.7 ± 0.6	2.5 ± 2.1
Aorta at diaphragm (*N* = 495) mean ± SD (mm)	26.4 ± 2.4	0.9 ± 0.9	3.3 ± 3.3
Heart (*N* = 337)			
Heart volume mean ± SD (mm³)	774.4 ± 169.8	59.0 ± 58.4	7.5 ± 6.4
Coronary artery calcium (*N* = 520)			
Volume score median (IQR**)	39.2 (11.8–143.6)	11.2 (4.2–29.2)	28.5 (11.3–69.5)

*SD* standard deviation, *IQR* interquartile range

### Agreement between baseline and repeat measurements

Excellent agreement between baseline and repeat measurements was found for vertebral height and density with ICC of 0.986 (95% CI: 0.983–0.988) and 0.996 (95% CI: 0.995–0.997), and for all diameter measurements of the thoracic aorta, with ICC ranging from 0.931 (95% CI: 0.918–0.942) at the position of the diaphragm to 0.987 (95% CI: 0.985–0.989) at the mid part of the ascending thoracic aorta. For heart volume and CACV, ICC was 0.938 (95% CI: 0.923–0.950) and 0.973 (95% CI: 0.968–0.977), respectively.

### Quantitative analysis of repeatability

Table 1 shows quantitative outcomes for cases in which baseline and repeat scan were considered adequate after visual evaluation, reporting mean diameter for baseline scan, mean absolute difference between baseline and repeat scan, and relative difference. Relative differences were small for AI-based vertebral analysis, < 3% for all tested parameters, indicating excellent repeatability between scans. For AI-based aortic analysis, relative differences remained < 4% for all measurement locations. Mean absolute differences in AI-based vertebral and aortic analysis were relatively small, and well within two standard deviations of the initial measurement at the baseline scan.

Relative difference for AI-based heart volume was ≥ 7.5%, indicating intermediate repeatability between baseline and repeat scan in visually assessed adequate cases.

AI-based CACV resulted in median absolute difference of 11.2 (IQR: 4.2–19.2), and relative difference of 28.5% (IQR: 11.3–69.5%). Supplementary Table 2 reports median CACV for cases in which AI-based analysis was adequate in either baseline or repeat scan, showing a high median CACV of 418.8 (IQR: 186.9–1094.2). This relatively high score indicates that inadequate AI-based CACV mostly appears in participants with a relatively large extent of CAC.

### Risk assessment based on CACV

Risk assessment for baseline and repeat AI-based CACV showed substantial agreement (kappa value of 0.71) (*p* < 0.001), see result in Table 2. Overall, risk classification between scans resulted in a concordance of 81.2%, with concordance per risk category ranging from 62.6% to 91.5%. In case of a difference in classification between baseline and repeat CT, 99.0% were categorized in the adjacent risk category.

**Table 2:** Confusion matrix of risk categories for baseline and repeat coronary artery calcium volume scoring

		Repeat scan (volume score)					Concordance
		< 10	10–99	100–499	≥ 500	Total	
Baseline scan (volume score)	< 10	**67**	40	0	0	107	67 (62.6%)
	10–99	28	**219**	14	1	262	219 (83.6%)
	100–499	0	6	**108**	4	118	108 (91.5%)
	≥ 500	0	0	5	**28**	33	28 (84.8%)
	Total	95	265	127	33	**520**	422 (81.2%)

Bold values represent the number of concordant cases in baseline and repeat scan

## Discussion

In 632 participants of a population-based study, we evaluated the repeatability of AI-based automated measurements in low-dose chest CT. Vertebral and aortic analysis showed accurate and highly repeatable results, with mean relative differences within < 4%. Heart segmentation following the pericardium is not yet optimized and resulted in overestimation of the heart volume. CACV showed adequate segmentations in 86.0% of cases when visually assessed. Although it resulted in moderate repeatability of CACV scores between scans, risk categorization for CACV nevertheless showed high overall concordance.

There are prior studies on validation of the AI-Rad Companion chest CT application, some with differences with the algorithm that we used since we applied a newer prototype version. We previously described the performance of thoracic aortic analysis in 240 ImaLife participants, showing mean absolute differences between a manual reader and AI within 2 mm for all measurement positions [9]. Van Assen et al published a validation study comparing automatic CACV on chest CT with manual scoring on cardiac CT in 263 patients, showing excellent correlation [14]. Additionally, Yang et al showed that AI-based vertebral density correlates strongly with bone marrow density values as obtained by dual-energy X-ray absorptiometry [15], indicating the possibility to detect a population at high risk of osteopenia with the use of chest CT.

However, in these studies, it remains unknown if AI-based results are reproducible in a scan-rescan setting. To our knowledge, no previous studies have evaluated the scan-rescan repeatability of vertebral, aortic and cardiac biomarkers on low-dose non-contrast chest CT using this AI software Somewhat in line with our aim, Graby et al evaluated the repeatability of aortic measurements applying the AI-Rad Companion chest CT algorithm to 25 separate contrast and non-contrast CT acquisitions resulting in moderate to good agreement (0.57–0.88) [16]. However, in this particular study, they used the same CT examination, which does not represent a test-retest setting since participants did not leave the CT table between acquisitions. Additionally, the comparison of scans with and without contrast agent might lead to the inclusion of the aortic wall in non-contrast-enhanced scans, resulting in an overestimation of the variability between scans.

In our study, vertebral and aortic analyses showed high accuracy when visually assessed (except for the aorta measurement at the sinotubular junction). In line with visual assessment, the mean discrepancy in AI-based vertebral and aortic measurements between scans was well within clinically relevant ranges. This would be at least > 50% height loss in at least one vertebra according to current recommendations for referral after low-dose chest CT screening [1]. Concerning the ascending thoracic aorta with diameter < 55 mm, a growth rate of 3 mm/year in 2 consecutive years or 5 mm in 1 year is an indicator of surgical intervention [9, 10]. High repeatability for these biomarkers indicates small error margins of AI between scans, with the potential to detect small changes in biomarker values as indicator of progression in follow-up scanning. This could especially lessen the burden for radiologists in a repeated opportunistic screening setting.

Visual evaluation of heart volume segmentation showed poor accuracy due to difficulty in tracing the pericardium. In inadequate segmentations, heart volume was overestimated due to the inclusion of paracardial fat. Additionally, heart volume showed moderate repeatability when only adequate segmentations were included. While interpreting these results, it should be taken into account that included chest CT scans were non-ECG-synchronized, potentially resulting in more discrepancy of AI-based heart volume between scans.

The non-ECG-synchronized characteristic of our chest CT scanning probably also influenced AI-based CACV, which showed poor repeatability in absolute measurements; thus, the poor repeatability is due to motion artifacts rather than suboptimal segmentation of AI. A previous study in the ImaLife cohort showed that low-dose, non-ECG-triggered chest CT scans could result in a slight underestimation of CAC score when compared to ECG-triggered cardiac CT scans. Xia et al found that non-ECG-triggered chest CT scans had a median percentage difference of 29% compared with cardiac CT scans [17]. Additionally, Šprem et al showed that scans containing CAC severely affected by motion artifacts demonstrate lower CAC score reproducibility [18]. We considered increased variability in CACV due to motion to be a consequence of the acquisition protocol and not a sign of inaccurate AI performance. This reasoning is further supported by the fact that when in our study only accurate cases were included for analysis (and suboptimal cases excluded), relative differences were still > 20%. The variability of CACV in our cohort with high-pitch chest CT is quite high and might even increase in standard single-source CT setting.

Visual assessment showed adequate segmentation by AI in 86.0% of cases. In most cases where AI was inaccurate, calcifications in the left main and left anterior descending artery were segmented too small. This might lead to misclassification of patients into lower-risk categories and therefore potentially influence clinical decisions and long-term outcomes. However, risk categorization in our study shows concordance in > 80% of adequately segmented cases and in case of difference in classification, 99.0% were categorized in the adjacent risk category. When looking at individual risk categories, concordance is relatively poor in the very low-risk category. As the range of CACV values in the low-risk category is relatively small, and the variability in chest CT is relatively high, any deviation in CACV can easily affect risk categorization. However, in a screening setting, it would be of greater importance to identify persons with a moderate or high risk of cardiovascular disease based on CACV. Golub et al argued that especially a cut-off value of ≥ 100 would be of interest to identify moderately to highly increased risk of cardiovascular disease [19]. Using this cut-off value leads to > 95% concordance for AI.

A strength of our study was our scan-rescan set-up, in which participants received a follow-up scan using the same high-pitch, low-dose CT scan protocol. Additionally, the set-up represented a follow-up protocol with a time interval between scans resembling clinical or screening scenario (in case of intermediate-size lung nodule) and new positioning of participants on the CT table. This mimics a protocol potentially to be used in low-dose chest CT lung cancer screening.

The retrospective, single-center, single-scanner protocol was also a limitation of our study. The results were limited to the population originating from the ImaLife study receiving a repeat scan, potentially leading to a selection bias with regard to the presence and distribution of chest imaging biomarkers. Our results cannot be extended to scans from other scanner vendors or other populations without further validation. Moreover, visual assessment was done by one trained, certified technical physician, so no consensus read was done. However, the reader was supervised by a board-certified ECBR radiologist with 15+ years of experience for inconclusive cases. Based on this visual assessment, our quantitative analysis only included adequately segmented cases, ignoring inadequate measurements. In clinical practice, this would mean that each case should still be supervised by humans to prevent under- or overtreatment of individuals participating in screening if the diagnosis would be solely based on AI. Even though human supervision remains needed, the burden for radiologists would be lessened by the automatic biomarker quantification in adequate cases.

In conclusion, segmentation accuracy of AI-based software in low-dose chest CT was high for vertebral, aortic and CACV evaluation and relatively low for heart volume. Repeatability of vertebral and aortic measurements was excellent. While repeatability for CACV was modest, concordance in risk categorization was high.

## Supplementary information

ELECTRONIC SUPPLEMENTARY MATERIAL

## Abbreviations

AI: Artificial intelligence
CABG: Coronary artery bypass graft
CAC: Coronary artery calcium
CACV: Coronary artery calcium volume
CT: Computed tomography
ICC: Intraclass correlation coefficient
ImaLife: Imaging in Lifelines
IQR: Interquartile range
PCI: Percutaneous coronary intervention
SD: Standard deviation
