# Supplementary material for: Repeatability of AI-based, automatic measurement of vertebral and cardiovascular imaging biomarkers in low-dose chest CT: the ImaLife cohort
Source: Eur Radiol. 2025 Jan 8;35(7):3833–41. doi: 10.1007/s00330-024-11328-9 (PMC12165965; doi:10.1007/s00330-024-11328-9)
Supplement: Supplementary file 1 — ELECTRONIC SUPPLEMENTARY MATERIAL [file 330_2024_11328_MOESM1_ESM.pdf]

Repeatability of AI-based, automatic measurement of vertebral and cardiovascular imaging biomarkers in low-dose chest CT: the ImaLife cohort

ELECTRONIC SUPPLEMENTARY MATERIAL

Eur Radiol (2024) Hamelink I, van Tuinen M, Kwee TC, van Ooijen PMA, Vliegenthart R.

Supplemental table 1 Criteria for grading of suboptimal or inaccurate segmentations and measurement of imaging biomarkers

Region	Suboptimal	Inaccurate
Vertebrae	• Incorrect numbering of vertebrae<br>• ROI for HU value off-centre within vertebrae, but still within vertebrae borders<br>• Small segmentation errors of vertebrae (<10% of total volume), but not impacting vertebral height measurements	• Incorrect height measurement, due to incorrect segmentation of vertebrae
Aorta	• Positioning of measurement within 1 cm of optimal measurement location as described by ESC/AHAA guidelines<br>• Suboptimal angulation of aortic measurement of <20 degrees of optimal angulation<br>• Small segmentation surface errors (<10%), but not impacting maximum diameter measurements	• Positioning of measurement > 1 cm of optimal measurement location as described by ESC/AHAA guidelines<br>• Suboptimal angulation of aortic measurement > 20 degrees of optimal angulation<br>• Significant segmentation surface errors impacting maximum diameter measurements
Heart	• Small segmentation errors including over- and underestimation of volume; <10% of total volume	• Segmentation errors including over- and underestimation of volume; >10% of total volume
Coronary artery calcium volume (CACV)	• Small spots erroneously detected as CACV, due to noise within the scan<br>• Small spots erroneously detected as CACV outside the pericardium<br>• Correctly identified calcified spots, but erroneously labeled coronary (e.g. spots within LM labeled as LAD)	• Inclusion of non-calcified spots as CACV<br>• Exclusion of calcified spots as CACV<br>• Inclusion of mitral valve calcification as CACV<br>• Inclusion of aortic calcification as CACV<br>• Inclusion of pericardial calcification as CACV<br>• Inclusion of bone as CACV

Supplemental table 2 AI-based quantitative assessment of vertebral and cardiovascular imaging biomarkers (with N>10) in low dose chest CT, mean value at baseline and absolute/relative difference between baseline and repeat CT for those with inaccurate segmentation at baseline or repeat CT scan

	**Correct AI-based analysis**	**Incorrect AI-based analysis**	**Absolute difference**	**Relative difference (%)**
Thoracic aorta (N=12-174)				
**Sinotubular junction (N=174) Mean ± SD* (mm)**	34.1 ± 4.1	34.3 ± 4.1	1.0 ± 1.1	2.9 ± 3.1
**Proximal arch (N=99) Mean ± SD (mm)**	33.9 ± 3.4	34.0 ± 3.3	0.9 ± 0.7	2.6 ± 2.3
**Mid arch (N=31) Mean ± SD (mm)**	32.4 ± 3.3	32.7 ± 3.4	0.8 ± 0.6	2.6 ± 2.0
**Proximal descending (N=12) Mean ± SD (mm)**	30.4 ± 2.9	30.8 ± 3.4	0.7 ± 0.7	2.2 ± 2.2
**Aorta at diaphragm (N=97) Mean ± SD (mm)**	26.9 ± 2.8	27.4 ± 2.8	1.2 ± 1.1	4.6 ± 4.2
Heart (N=64)				
**Heart volume Mean ± SD (mm³)**	933.3 ± 239.7	958.6 ± 249.8	63.8 ± 46.2	7.2 ± 5.1
Coronary artery calcium (N=45)				
**Volume score Median (IQR**)**	418.8 (186.9 – 1094.2)	392.7 (138.9 – 776.4)	112.9 (39.5 – 241.9)	25.5 (11.1 – 59.4)

*SD = standard deviation; **IQR = interquartile range

Supplemental Figure 1 Examples of suboptimal classified segmentations for vertebral, aortic and cardiac biomarkers which were included for further analysis including ICC, assessment of relative differences, and risk categorization for CACV.

a) Incorrect numbering of vertebrae, and suboptimal positioning of density ROI at T3 level (red, circled). b) Suboptimal angulation and positioning of thoracic aortic diameter measurements at the sinotubular junction and proximal aortic arch (red, circled), respectively. c) Suboptimal segmentation of pericardium (true pericardium indicated with red arrows), leading to minimal overestimation and underestimation of heart volume, respectively. d) Inclusion of noise as CACV within right coronary artery and segment of a pulmonary artery volume as CACV within circumflex coronary artery (red, circled).

(a)

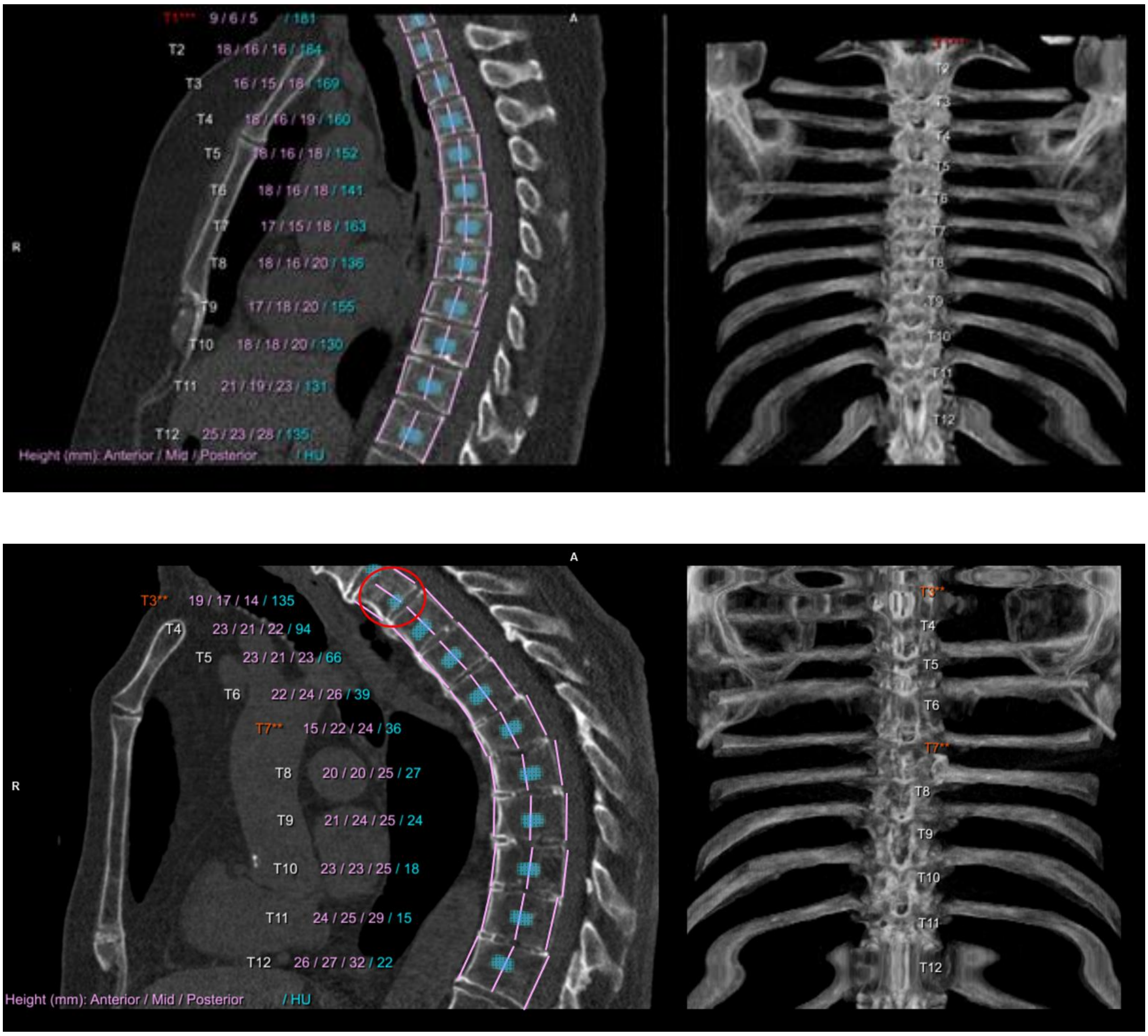

(b)

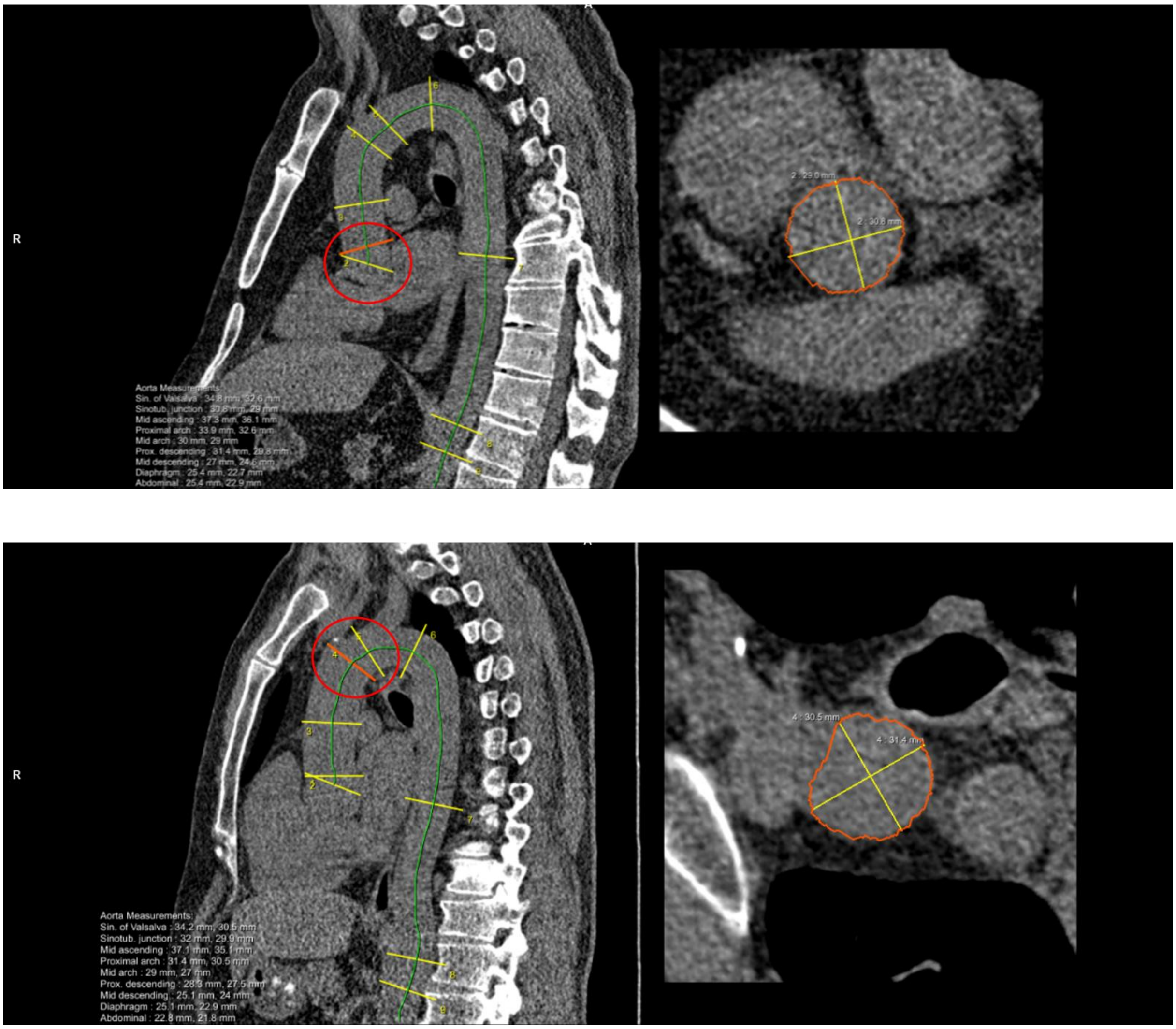
R
Aorta Measurements:
Sin. of Valsalva : 34.8 mm, 32.6 mm
Sinotub. junction : 30.8 mm, 29 mm
Mid ascending : 37.3 mm, 36.1 mm
Proximal arch : 33.9 mm, 32.6 mm
Mid arch : 30 mm, 29 mm
Prox. descending : 31.4 mm, 29.8 mm
Mid descending : 27 mm, 24.6 mm
Diaphragm : 25.4 mm, 22.7 mm
Abdominal : 25.4 mm, 22.9 mm
2 : 29.0 mm
2 : 30.8 mm
R
Aorta Measurements:
Sin. of Valsalva : 34.2 mm, 30.5 mm
Sinotub. junction : 32 mm, 29.9 mm
Mid ascending : 37.1 mm, 35.1 mm
Proximal arch : 31.4 mm, 30.5 mm
Mid arch : 29 mm, 27 mm
Prox. descending : 28.3 mm, 27.5 mm
Mid descending : 25.1 mm, 24 mm
Diaphragm : 25.1 mm, 22.9 mm
Abdominal : 22.8 mm, 21.8 mm
4 : 30.5 mm
4 : 31.4 mm

(c)

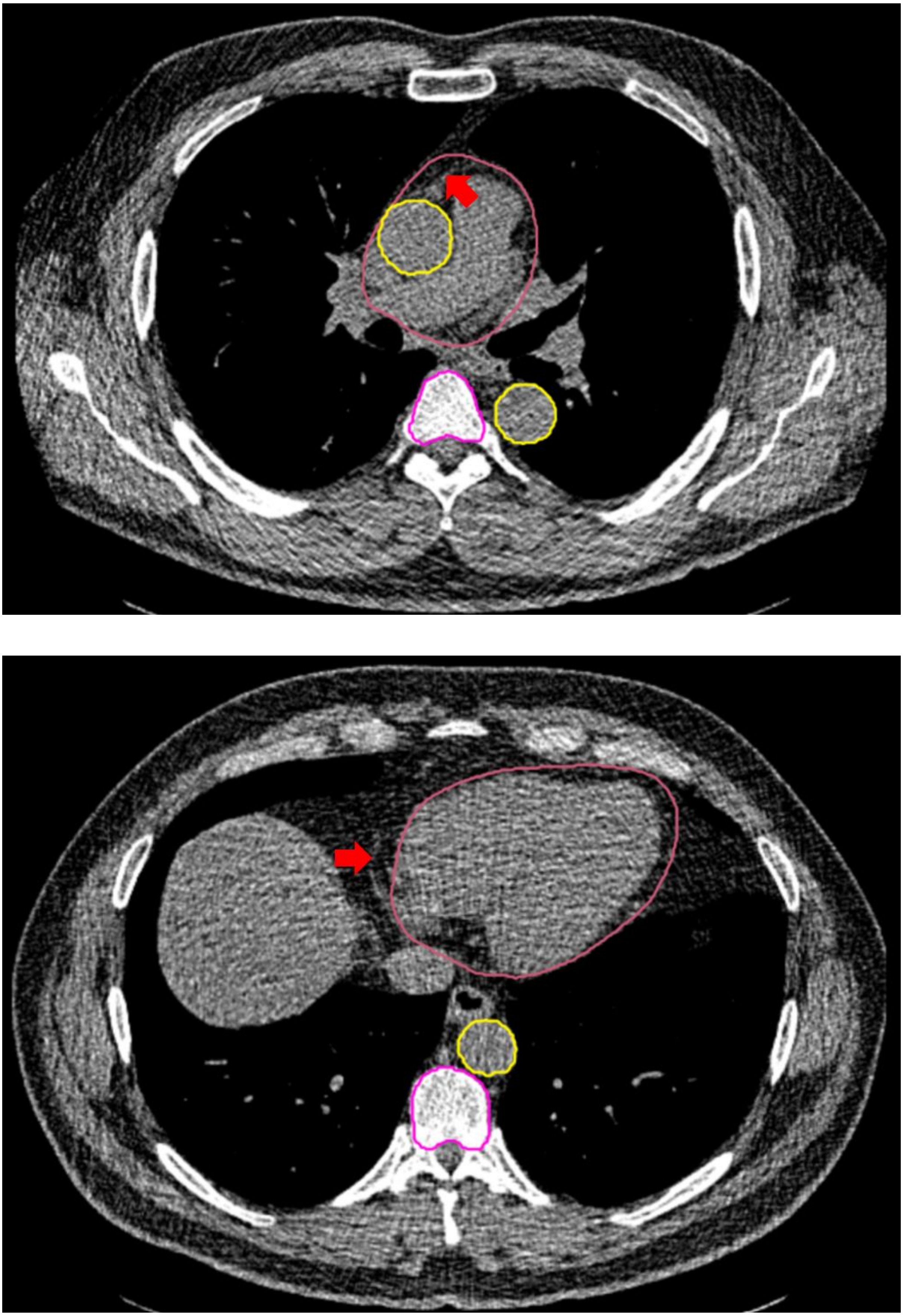

Eur Radiol (2024) Hamelink I, van Tuinen M, Kwee TC, van Ooijen PMA, Vliegenthart R.

(d)

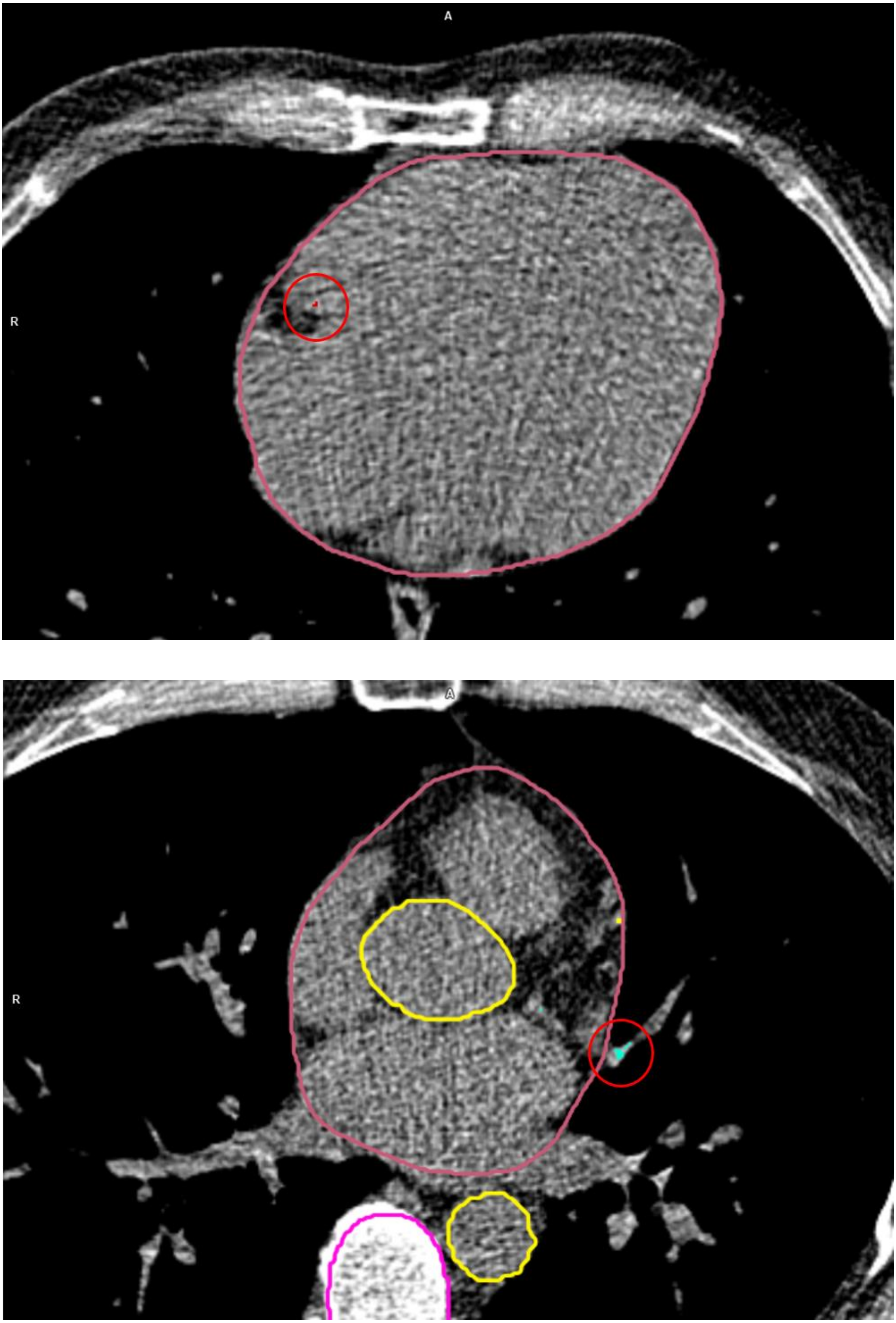

Eur Radiol (2024) Hamelink I, van Tuinen M, Kwee TC, van Ooijen PMA, Vliegenthart R.
